# CDC Activities to Enhance Training in Cancer Prevention and Control in Field Epidemiology Training Programs in Low- and Middle-Income Countries

**DOI:** 10.1200/JGO.18.00042

**Published:** 2018-05-14

**Authors:** Virginia Senkomago, Rachael Joseph, Monica Sierra, Elizabeth Van Dyne, Meheret Endeshaw, Denise Duran, David E. Sugerman, Mona Saraiya

**Affiliations:** **Virginia Senkomago**, **Rachael Joseph**, **Elizabeth Van Dyne**, **Meheret Endeshaw**, **Denise Duran**, **David E. Sugerman**, and **Mona Saraiya**, Centers for Disease Control and Prevention, Atlanta; and **Monica Sierra**, Task Force for Global Health, Decatur, GA.

## Abstract

Cancer is one of the leading causes of morbidity and mortality worldwide. In 2012, there were > 14 million new cancer cases and > 8 million cancer deaths, with 70% of these deaths occurring in low- and middle-income countries (LMICs). Part of the success of cancer prevention and control efforts requires the development and strengthening of the public health workforce, particularly in LMICs where the cancer burden is the greatest. The US Centers for Disease Control and Prevention (CDC) supports workforce capacity development globally through Field Epidemiology Training Programs (FETPs) established in ministries of health in > 70 countries. To enhance training in cancer prevention and control in FETPs, the CDC has developed an open-access curriculum in applied cancer epidemiology and supports FETP trainees who conduct cancer-related planned projects. The curriculum contains modules on cancer registration, screening, and comprehensive cancer control that are particularly relevant to current cancer control efforts in many LMICs. Pilot testing of the curriculum showed an increase in trainees’ cancer knowledge and covered content trainees found to be relevant to their field epidemiology training and projects and future work in cancer prevention and control. Since 2013, the CDC has supported 13 trainees with cancer-related projects; two have published articles, two have presented their results at international conferences, and others are writing manuscripts on their project outcomes. Through the development of an open-access applied cancer epidemiology curriculum and by supporting cancer-related projects for FETP trainees, the CDC provided technical assistance for LMICs to build capacity for cancer prevention and control efforts.

## INTRODUCTION

Noncommunicable diseases (NCDs) are responsible for approximately 70% of deaths worldwide, and cancer is the second largest contributor to NCD deaths.^1^ In 2012, there were an estimated 14.1 million new cancer cases and 8.2 million cancer deaths worldwide, with the majority of cancer cases (57%) and deaths (65%) occurring in less-developed regions.^2^ Global cancer incidence is projected to increase to 20 million by 2025, and the greatest incidence increase is expected in low- and middle-income countries (LMICs).^3^ With the growing cancer burden, part of the success of public health efforts in cancer prevention and control will require workforce development, particularly in LMICs where the cancer burden is the greatest.^4^

For > 40 years, the US Centers for Disease Control and Prevention (CDC) has supported workforce capacity development globally through the Field Epidemiology Training Program (FETP).^5^ The 2-year FETP in applied epidemiology is modeled after the CDC’s US-based Epidemic Intelligence Service training program and currently is established in > 70 countries.^6^ FETP trainees are positioned within ministries of health, and the majority of their training involves fieldwork under the supervision and mentorship of trained field epidemiologists. The FETPs in LMICs have focused primarily on training field epidemiologists in the prevention and control of infectious diseases that are still highly prevalent in many of these countries. However, many LMICs are now experiencing similar or greater deaths from NCDs and infectious diseases and are looking to the FETPs in their countries to train field epidemiologists for NCD prevention and control.^7^

The CDC has supported FETPs in conducting NCD training since 2010 with an approach that focuses on developing curricula and training materials, providing small awards to trainees for planned projects and fieldwork, providing mentorship, and providing support for NCD-focused FETP resident and fellowship positions.^8^ Since 2013, the CDC’s Division of Cancer Prevention and Control has been working with FETPs in LMICs to support the training of field epidemiologists in cancer prevention and control. This report describes the work of the Division of Cancer Prevention and Control in developing an open-access applied cancer epidemiology curriculum to train field epidemiologists and providing awards and mentorship for planned projects to FETP trainees in several countries.

## CDC APPLIED CANCER EPIDEMIOLOGY CURRICULUM

### Development of the Curriculum

The CDC developed an applied cancer epidemiology curriculum to create a comprehensive open-access resource for training field epidemiologists in applied cancer prevention or control programs. Topics covered in the curriculum were chosen on the basis of their relevance to current cancer control efforts in limited-resource settings. We also chose to focus on cancer control approaches in which the CDC has extensive experience and demonstrated success in the Unites States. The four topic areas currently covered in the curriculum are introduction to cancer epidemiology prevention and control, comprehensive cancer control, principles of cancer registries, and principles of cancer screening programs (Table 1).

**Table 1:**
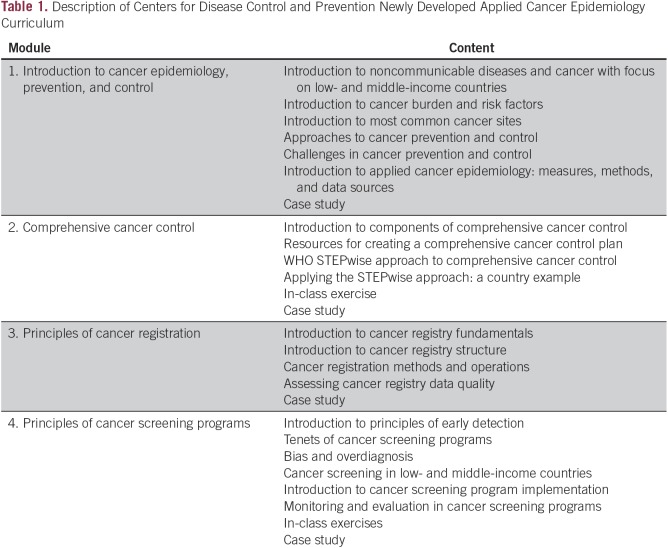
Description of Centers for Disease Control and Prevention Newly Developed Applied Cancer Epidemiology Curriculum

CDC experts in collaboration with the Training Programs in Epidemiology and Public Health Interventions Network (TEPHINET) developed the content for the curriculum. Cancer experts at various national and international organizations, including the US National Cancer Institute, the International Agency for Research on Cancer (IARC), and the Karolinska Institute, reviewed the draft curriculum and modules. Feedback from expert reviewers was used to guide revisions of the modules before pilot testing. The curriculum content comprises slide presentations; facilitator notes; accompanying reading material; and interactive learning activities, including in-class exercises, examples, and case studies. The draft curriculum was pilot tested with FETP trainees from several countries before finalizing the content.

### Curriculum Pilot Test

In-person trainings were conducted with three modules of the draft curriculum (introduction to cancer epidemiology, cancer screening, and cancer registration) to assess the utility and presentation of the content and to inform revisions for the final curriculum. Modules of the draft curriculum were pilot tested in collaboration with various partners at four workshops: a 2-day workshop at an NCD-FETP training in Atlanta, Georgia (August 2015); a 2-day workshop at an FETP training held in collaboration with Tata Memorial Hospital in Mumbai, India (October 2015); a 3-day workshop at a cancer research training at the Sidi Mohammed Ben Abdellah University in Fes, Morocco (November 2015); and a 2-day workshop at the African Field Epidemiology Network Scientific Conference in Abuja, Nigeria (August 2016).

To improve the draft curriculum, questionnaires were administered to the FETP trainees before and after each training to assess their cancer knowledge for curriculum modules covered. The questionnaires obtained feedback on the relevance of the curriculum to trainees’ work and trainees’ opinions on the strengths and weakness of the content. FETP trainees ranked their knowledge before and after the training on a scale of 1 (lowest) to 5 (highest) for key cancer topics covered in the curriculum. The average knowledge scores for each cancer knowledge item and associated 95% CI from the pre- and post-training questionnaires were calculated in SAS statistical software (SAS Institute, Cary, NC).

One hundred twenty-three FETP trainees attended the four curriculum pilot testing workshops. These trainees were from 24 LMICs in Africa, Asia, the Middle East, and Latin America; the majority were from Nigeria (42%), Morocco (18%), and India (13%), where the training workshops were held. Ninety percent of the trainees had completed masters, doctoral, or medical school training before enrollment; however, 62% reported never having training or classes related to cancer prevention and control. Sixty-four percent of trainees had been working as physicians and 14% as public health professionals, including epidemiologists and environmental officers.

Results from the pre- and post-training questionnaires showed that the FETP trainees’ knowledge significantly increased for all the modules and topic areas after training with the draft curriculum; 95% CIs for average knowledge scores before and after training did not overlap for the cancer knowledge items assessed (Table 2). Trainees’ average score for self-reported knowledge in cancer epidemiology increased from 2.9 (95% CI, 2.7 to 3.1) to 4.3 (95% CI, 4.1 to 4.4) after the training. Similarly, trainees’ self-reported knowledge in cancer registration increased from 2.3 (95% CI, 1.9 to 2.6) to 4.0 (95% CI, 3.8 to 4.3) after the training, and knowledge on cancer screening increased from 2.3 (95% CI, 1.7 to 2.9) to 4.1 (95% CI, 3.9 to 4.3). All the trainees reported that the content of the curriculum was relevant to them and recommended the addition of more examples to these modules. Revisions were made to the draft curriculum to include more examples and in-class exercises in the finalized modules.

**Table 2:**
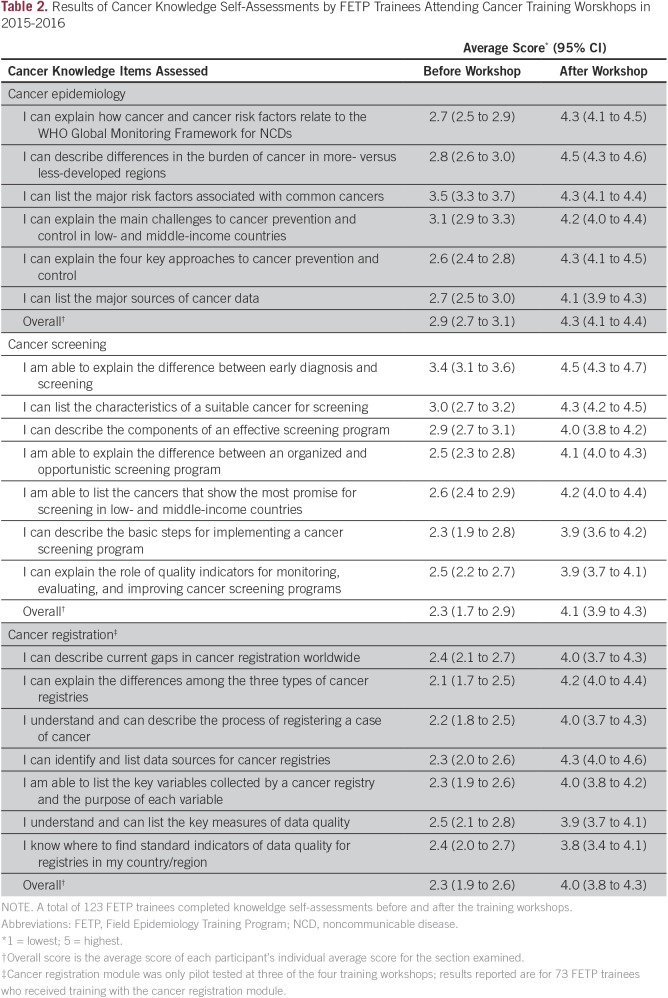
Results of Cancer Knowledge Self-Assessments by FETP Trainees Attending Cancer Training Worskhops in 2015-2016

### Description of Curriculum Modules

The four modules of this applied cancer epidemiology curriculum introduce the scientific background of each topic and provide a detailed practical examination of the topic through examples, in-class exercises, and case studies (Table 1). Each module takes approximately 10 hours to complete, thus 40 hours are required to complete the entire training. The content of each module stands alone so that trainees can focus on the modules most relevant to their cancer work. For trainees who are new to cancer control programs or research, training with the complete curriculum provides a detailed introduction to applied cancer epidemiology, comprehensive cancer control, cancer registration, and cancer screening programs.

#### Module 1: Introduction to Cancer Epidemiology, Prevention, and Control

This module provides a detailed introduction to the state of cancer prevention and control globally. It examines cancer risk factors, global cancer burden, and differences in cancer burden for various cancer sites in high-income countries versus LMICs. Trainees are provided with a practical examination of the challenges and complexities in estimating cancer burden and implementing cancer control measures, specifically in limited-resource settings. The module also discusses the methods and data sources that can be used to obtain good estimates for cancer awareness, cancer burden, and effectiveness of cancer control measures as well as clinical information on most common cancers. Examples and in-class exercises emphasize the challenges in addressing competing demands in cancer prevention and control with limited resources and examine notable success in cancer control worldwide. The case study walks trainees through the process of making data-driven decisions for cancer prevention and control in a limited-resource country and of making the case for prioritization of cervical cancer prevention amid competing cancer control priorities.

#### Module 2: Comprehensive Cancer Control

This module introduces trainees to the concepts of comprehensive cancer control planning, including the four key components of cancer control plans, the role of comprehensive cancer control programs, and the need for building partnerships in cancer control planning efforts. It discusses the process of developing a comprehensive cancer control plan by using the WHO STEPwise approach to comprehensive cancer control^9^ and examines one low-income country’s experience in applying this approach. Finally, the module discusses the need for evaluation of cancer control plans and programs and examines a program evaluation example that used the CDC’s framework for evaluation of comprehensive cancer control programs.^10^ The examples in the module discuss cancer control plans from LMICs and high-income countries and the use of cancer control plans to build partnerships and support cancer control in one middle-income country. Two case studies are included: one that focuses on the development of appropriate objectives and priorities for a comprehensive cancer control plan and a second that evaluates the implementation of a cancer control plan in an LMIC.

#### Module 3: Principles of Cancer Registration

This module examines the fundamentals of cancer registration and the complexities of obtaining high-quality cancer incidence and mortality data from cancer registries globally. It was developed in collaboration with experts at WHO/IARC and draws from the IARC summer course on cancer registration.^11^ The module discusses the various types of cancer registries (population, hospital, and pathology based), the key variables registries collect, the intricacies of the cancer registration process, and the global disparities in cancer registration. It examines how to monitor and evaluate the quality of cancer registry data and illustrates how data quality measures can be tracked with WHO/IARC CanReg software.^12^ The module also contains a section on hospital-based cancer registries, the most common type of registries and a starting point to build a population-based cancer registry in many LMICs. The collection, quality control, analyses, and utility of hospital-based cancer registry data are discussed, with examples from registries in several countries. Finally, the module examines how various analyses of cancer registry data can inform health care planning as well as assess the effect of cancer prevention and control interventions, cancer treatment, and patient care.

The module contains examples from > 12 cancer registries in high-income countries and LMICs. The in-class exercises focus on learning how to assess data quality by using existing registry data and to examine how cases are assigned and entered into various registries through de-identified summaries. The case study for this module evaluates cancer registration in a low-income country. Trainees are tasked with assessing the strengths and weakness of the described population-based cancer registry and to suggest possible recommendations for strengthening the cancer registry.

#### Module 4: Principles of Cancer Screening Programs

This module provides a comprehensive examination of cancer screening as a key approach to cancer prevention and control. It covers the key tenets of cancer screening, including the types of cancer suitable for screening and the need for appropriate screening tests and effectively organized screening programs. This module examines the components of effective cancer screening programs and discusses the data sources and key indicators to focus on in monitoring and evaluating them. Also discussed are the possible harms of screening, including overdiagnosis, and the biases to consider while examining data to assess the effectiveness of screening programs (selection bias, lead-time bias, length bias). Finally, this module examines the challenges in implementing cancer screening in LMICs and discusses the recommended strategies for screening and treatment of some of the most common cancers in LMICs (cervical, breast, colorectal, oral, and stomach). This module includes examples of cancer screening programs and outcome data from > 11 countries. In-class exercises focus on assessing the suitability for cancer screening for various cancer types in different settings on the basis of incidence and survival rates for the cancers and consideration of the available screening tests, treatment, and primary prevention strategies available in each setting. An exercise is included that examines various trends in cancer outcomes and effects of changes in cancer screening availability or cancer registration practices. The case study walks trainees through the process of making data-driven decisions for a cervical cancer screening strategy in a low-income country that is based on the cancer burden, existing resources, and current prevention and control efforts. Trainees review data from the cancer registry and a population-based survey conducted in the country, are provided with information about current cervical cancer efforts and resources, and have to provide recommendations on a cervical cancer screening strategy to the country’s ministry of health.

The finalized curriculum with all four modules will be housed online on the TEPHINET website (http://tephinet.org/data-action-curriculum-cancer-prevention-and-control-low-and-middle-income-countries) to allow trainees to conduct self-guided learning or instructors to access the content to deliver in-person trainings. The curriculum initially will be available in English and Spanish and may be translated into other languages at a later date. More modules that explore advanced topics in applied cancer control also may be added to the curriculum.

## CDC SUPPORT FOR FETP TRAINEE PLANNED PROJECTS ON CANCER PREVENTION AND CONTROL

As previously described, trainees in CDC-supported FETPs spend the majority (75%) of their time in the conduct of fieldwork, with a requirement to conduct a planned project before completion of their training. In many LMICs, FETPs interested in conducting cancer-related projects have reported several challenges, including a lack of cancer control program or registry data for projects, lack of funds to embark on field projects, and limited availability of mentors with experience in conducting cancer-related projects. Since 2013, the CDC has supported FETP trainees with their cancer-related projects by providing small awards to those with competitive project proposals and collaborating with in-country FETP resident advisors to mentor trainees in completing the projects. Thirteen FETP trainees from eight LMICs have received mentoring or awards from CDC to conduct cancer-related projects (Table 3). Of these projects, five focused on analyzing or evaluating cancer registry data; two on evaluated cancer screening programs; two assessed the implementation of cancer policies; and four analyzed survey data to evaluate knowledge, attitudes, and practices for cervical cancer screening among women and health care providers. All seven FETP trainees whose cancer-related planned projects were supported between 2013 and 2016 have completed their projects: two published articles,^13,14^ two presented their results at international conferences,^15,16^ and three are working on manuscripts. These FETP graduates are able to apply and build on the cancer knowledge and public health skills they gained through their FETP training while working in their current positions in local NCD departments, medicine, and academia. In addition, two FETP projects, the assessment of cancer registry data at the Thailand National Cancer Institute and the evaluation of the referral mechanisms for breast and cervical cancer screening in Tamil Nadu State, India, provided pilot data that have informed current research initiatives.

**Table 3:**
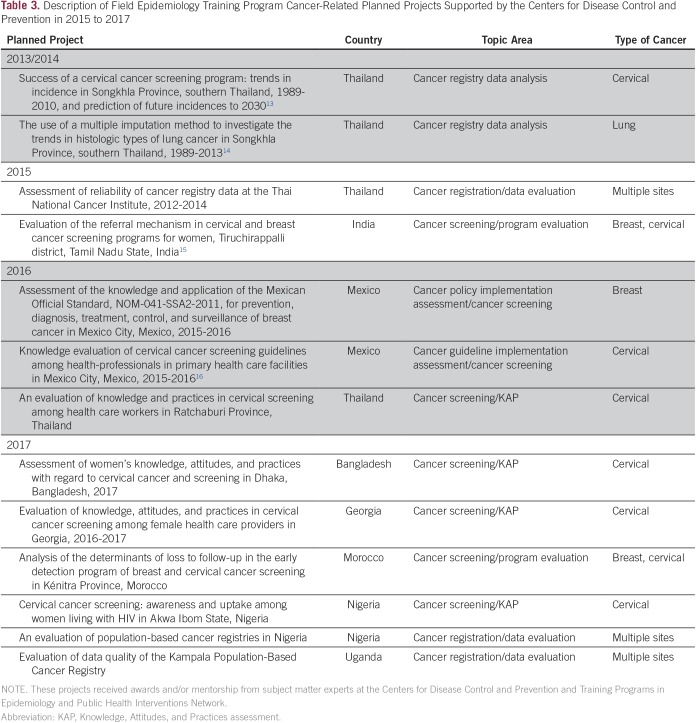
Description of Field Epidemiology Training Program Cancer-Related Planned Projects Supported by the Centers for Disease Control and Prevention in 2015 to 2017

In conclusion, the growing cancer burden in LMICs necessitates a trained public health workforce to implement cancer prevention and control efforts in these countries. The CDC has supported the training of field epidemiologists through the FETP in > 70 countries for > 40 years. Whereas the main focus of most FETPs is to train field epidemiologists in infectious disease and outbreak prevention and control, the growing burden of NCDs, including cancer, highlights the need to train LMIC field epidemiologists in NCD prevention and control.

The CDC has developed an applied cancer epidemiology curriculum to provide FETP trainees with the knowledge to contribute to cancer prevention and control efforts in their countries. It focuses on cancer registration, screening, and comprehensive cancer control, which are particularly relevant in many LMICs where these cancer control and surveillance efforts are still nascent. The pilot test revealed an increase in FETP trainees’ cancer knowledge and that the curriculum covered content the trainees found to be relevant to their field epidemiology training, cancer-related projects, and future work in cancer prevention and control. The curriculum will be published online to provide a free comprehensive resource for training in applied cancer epidemiology for FETP trainees and public health officials worldwide.

In addition to this curriculum, the CDC builds capacity for cancer prevention and control in several LMICs by supporting FETP trainees in conducting cancer-related planned projects. Since 2013, 13 FETP trainees have conducted cancer-related projects supported by awards and/or mentorship. These projects provide hands-on experience that enables trainees to work closely and build relationships with cancer experts in ministries of health or national cancer institutes. Cancer-related projects conducted by FETP trainees have answered key questions on applied cancer control efforts and provided pilot methodology and data for research studies and activities in cancer prevention and control in some countries. Through the development of an open-access applied cancer epidemiology curriculum and by supporting cancer-related projects for trainees in FETPs, the CDC enables LMICs to build capacity for cancer prevention and control efforts.
